# Norwegian hourly residential electricity demand data with consumer characteristics during the European energy crisis

**DOI:** 10.1016/j.dib.2023.109687

**Published:** 2023-10-14

**Authors:** Matthias Hofmann, Sigurd Bjarghov, Stian Nessa

**Affiliations:** a Statnett SF, Nydalen allé 33, 0484 Oslo, Norway; b Department of Electric Power Engineering, NTNU, O. S. Bragstads plass 2E, 7034 Trondheim, Norway; c Department of Energy Systems, SINTEF Energy Research AS, Sem Sælands vei 11, 7034, Trondheim, Norway

**Keywords:** Electricity demand, Survey, Households, Demand flexibility

## Abstract

This dataset was collected to understand how Norwegian households responded to the electricity price shock due to the European energy crisis. It consists of consumer characteristics and their self-reported responses to the extraordinarily high electricity prices which were collected with a survey of 4,446 consumers. The consumer characteristics contain socio-demographic information, such as income, age, education, number of residents, residence type, residence size, and how conscious the respondents were about their electricity consumption. Furthermore, major electricity-consuming appliances were identified, such as whether the residents had an electric vehicle and how they heated their homes, and if they had a dynamic electricity price contract. In addition, the dataset includes hourly metered electricity consumption data covering October 2020 to March 2022 from a subset of 1,136 residential consumers of the surveyed households, the total hourly residential electricity consumption per Norwegian bidding area from July 2019 to July 2022, and the hourly day-ahead electricity prices. These data are interesting to researchers that aim to gain insight into the electricity consumption behaviour of the residential sector and the impact of different socio-demographic variables.

Specifications Table

**Table:** 

Subject	Energy economics
Specific subject area	Electricity load profiles and the price responsiveness of households
Type of data	Table (csv files)
How the data were acquired	The survey answers were gathered through online questionnaires. Electricity consumption data were gathered through smart meters and made available by the local grid companies and the national electricity consumption data platform Elhub.
Data format	Raw Filtered
Description of data collection	The online survey was conducted by the market research company Ipsos with a pre-recruited household panel and was limited to the regions of Oslo, Bergen, Trondheim, and Tromsø. Hourly electricity consumption data were collected via local grid companies from a subset of the surveyed households. These households consented to share their data in the survey, and a matching entry based on name and address was found in the database of the local grid company for them. Households with consumption patterns that could be identified as outliers were excluded. Aggregated consumption data of all households per bidding area were collected from Elhub, the national database for electricity data.
Data source location	Survey answers: • Region: Oslo, Bergen, Trondheim, Tromsø • Country: Norway Electricity consumption on the household level: • Region: Oslo, Bergen • Country: Norway Electricity consumption on the bidding area level: • Region: NO1, NO2, NO3, NO4, NO5 • Country: Norway Electricity price: • Region: NO1, NO2, NO3, NO4, NO5 • Country: Norway • Data sources: Nordpool, ENTSOe Transparency platform, Norges Bank
Data accessibility	Repository name: Zenodo Data identification number: 10.5281/zenodo.8423312 Direct URL to data: https://zenodo.org/record/8423312

## 1 Value of the Data

• This dataset comprises a substantial amount of hourly metered electricity demand data spanning up to 36 months from households with a large share of dynamic electricity price contracts tied to the hourly spot price. These contracts, alongside the extraordinarily high electricity prices during the observation period, enable an analysis of demand flexibility in households influenced by variable prices.
• The dataset encompasses the highest recorded national peak load in Norway to date, rendering it particularly interesting for power system analysis and understanding the contributions of households to peak demand.
• The data presented in this article establish a connection between electricity demand data to consumer characteristics obtained through surveys. These characteristics include socio-demographic variables, households' attitudes and awareness towards their electricity consumption, their primary electricity-consuming appliances such as electric vehicles, and how they responded to extraordinarily high electricity prices. Consequently, these data facilitate investigation of household subgroups.
• The data were collected from a real-life context and closely resemble another dataset acquired during a dynamic pricing experiment involving households in the same regions [1]. Thus, the data facilitate comparison of the price responsiveness of households in experimental and real-life settings.
• The dataset can be employed to conduct socio-economic analyses required for policymakers to make informed decisions. Economists can explore fairness and welfare distributions of electricity costs for different consumer groups, whereas power system researchers can leverage the dataset to gain insights into price elasticity and demand patterns across consumer groups with different appliances and habits. Additionally, it enables a comparison of the stated preference and revealed preference for responding to high electricity prices.

## 2 Objective

The data collection was part of the iFlex project by the Norwegian transmission operator Statnett with the goal of investigating whether, and how, households responded to the extraordinarily high electricity prices in winter 2021/22 [2]. Most Norwegian households have electricity contracts tied to the hourly spot price for their bidding area and thus were exposed directly to these prices.

## 3 Data Description

We have divided the data description into two sections. The first describes the nature of the data, whereas the second describes the structure of the table files.

### 3.1 Nature of collected data

**Fig. 1:**
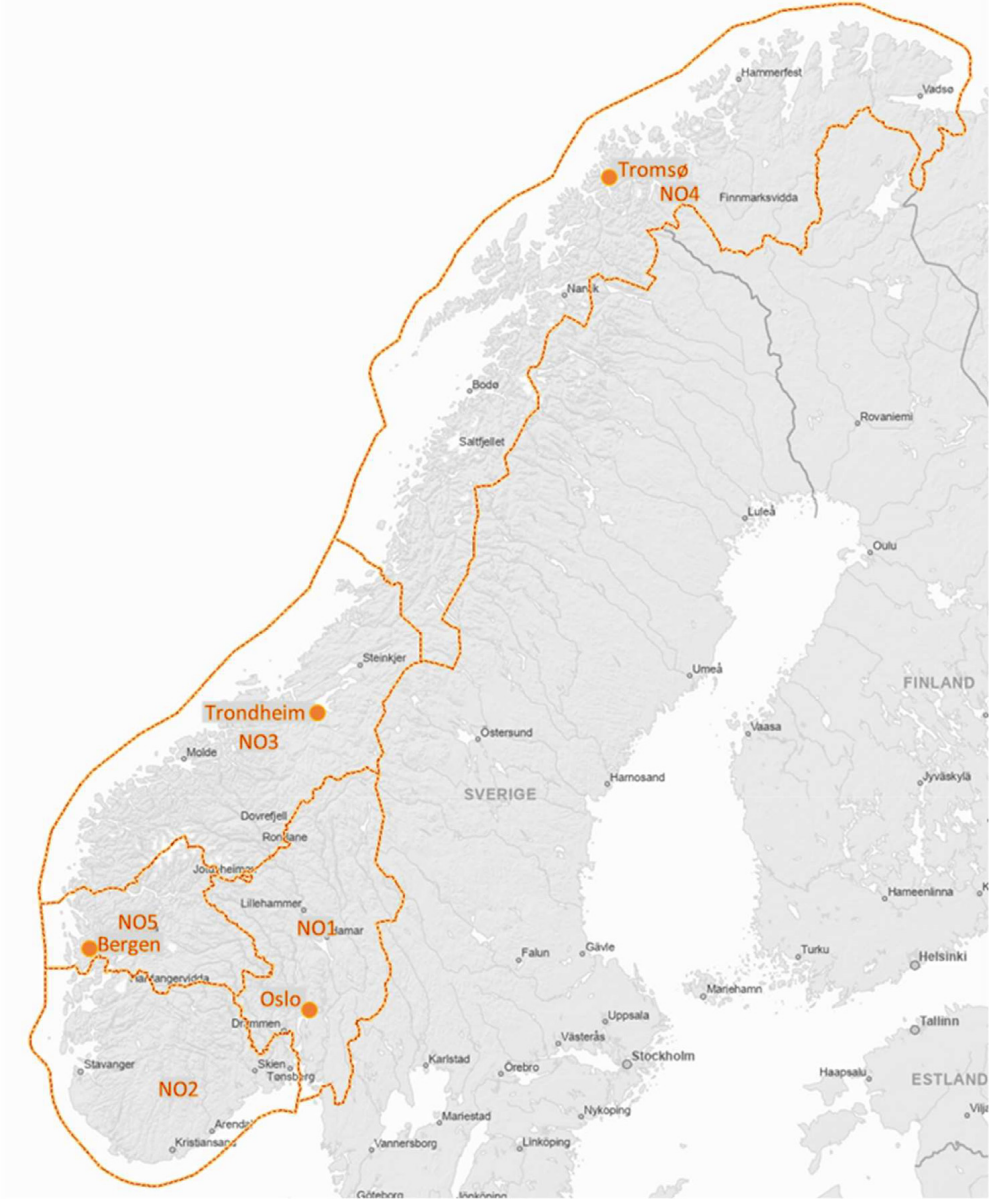
Location of the regions and bidding areas where the data were collected (map is published under Norwegian License for Open Government Data at https://temakart.nve.no/tema/nettanlegg).

The dataset contains four data types: survey answers, individual electricity consumption data, aggregated electricity consumption data, and electricity prices. Norway is divided into five bidding areas, with hourly electricity prices determined by the day-ahead market of Nordpool^1^ based on the production, consumption, and exchange capacities for each bidding area. The dataset covers these bidding areas, as summarised in Fig. 1. The following sections describe each data type.

#### 3.1.1 Survey answers

The survey was conducted in four different Norwegian regions, and the number of households answering it is summarised in Table 1.

**Table 1:** The number of survey answers per region and city.

Region	City	Number of household answers
Oslo		2,277
	Asker	96
	Bærum	198
	Lillestrøm	200
	Oslo	1,783
Bergen		994
Trondheim		385
Tromsø		790
Sum		4,446

**Fig. 2:**
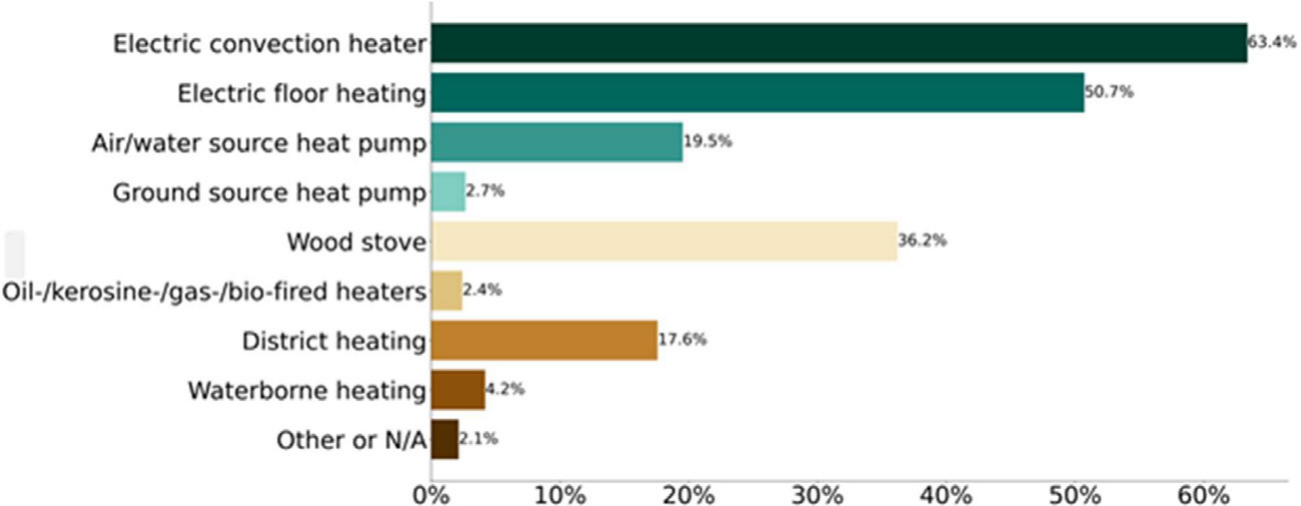
Share of households with specific heating source (multi-select multiple choice). N=4,446.

**Fig. 3:**
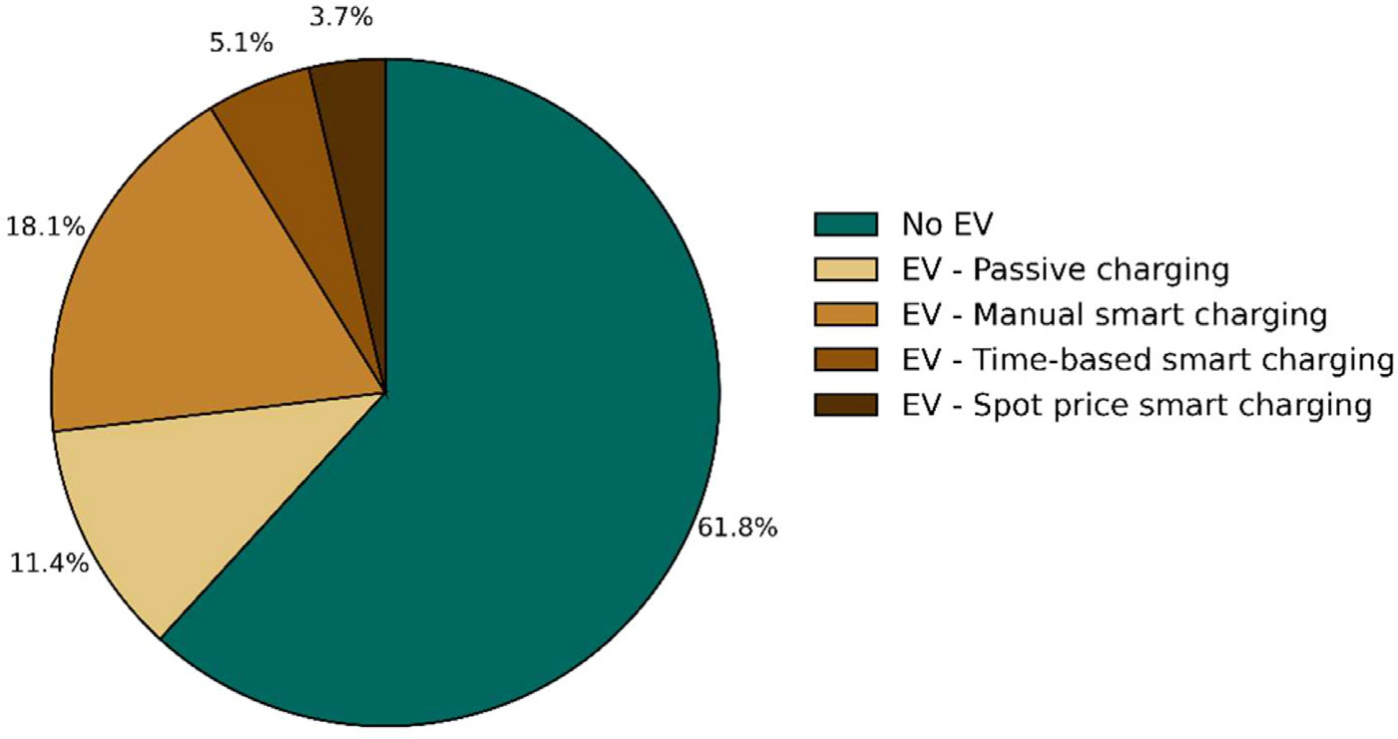
Share of households with an electric vehicle and how they charge it. N=4,446.

The survey contained questions to provide insight into whether the households had reduced their electricity consumption due to the high electricity prices that occurred in the previous winter. Additionally, questions were asked about the customers' type of electricity price contract, electricity consumption and costs awareness, and residence characteristics. These characteristics include building type, size, heating sources, and electric vehicle ownership. Finally, socio-demographic variables such as the number of inhabitants, income, age, and others were also included in the survey. A comprehensive list of the survey questions is provided in Section 4.1. The households included in the dataset exhibit two interesting characteristics: a high share of electric heating and electric car owner ship, as illustrated through the survey answers in Fig. 2 and Fig. 3.

#### 3.1.2 Individual electricity consumption

**Fig. 4:**
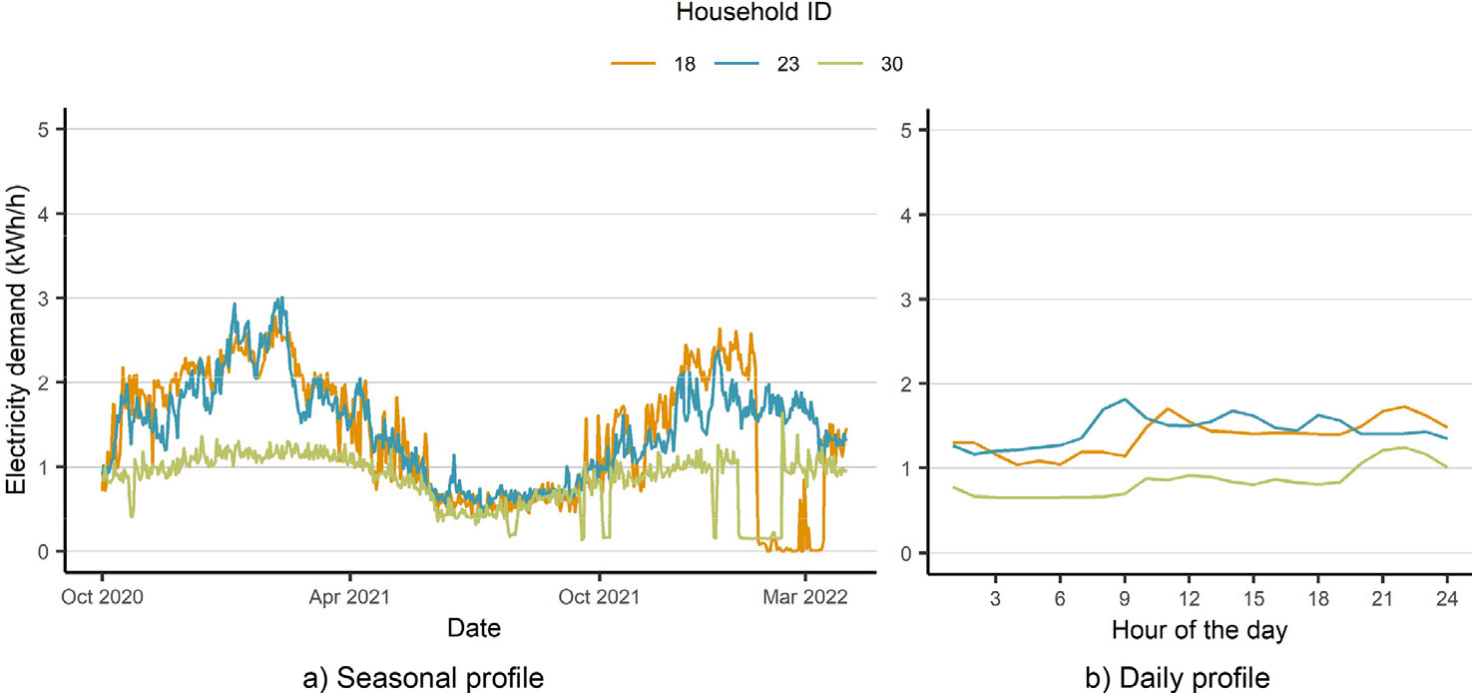
Seasonal (a) and daily (b) demand profiles of three individual households.

The dataset includes the hourly electricity consumption data of 1,136 households from two regions, Oslo, and Bergen, from October 2020 to March 2022. Examples of the available demand data of individual households are shown in Fig. 4.

#### 3.1.3 Aggregated electricity consumption

**Fig. 5:**
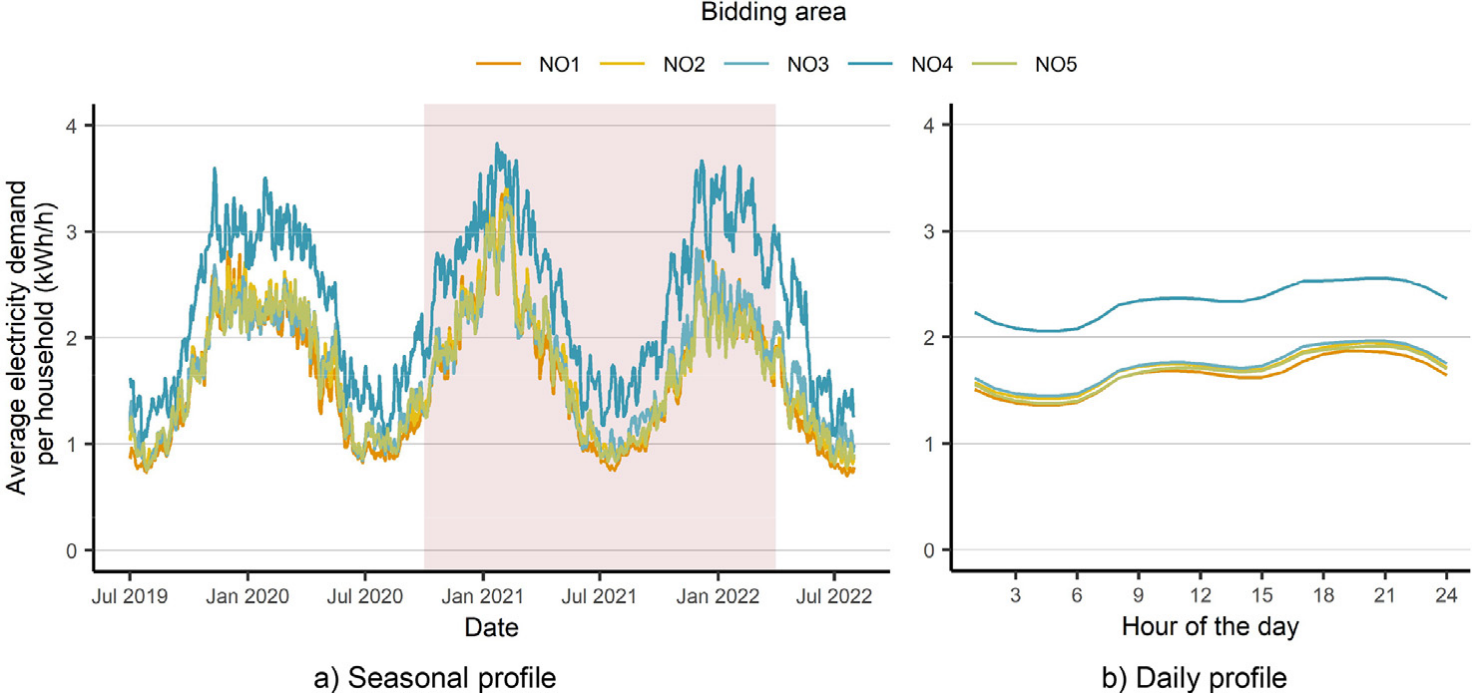
Seasonal (a) and daily (b) demand profiles of an average household in each bidding area based on the aggregated demand data and the number of residential electricity meters. The period which overlaps with the individual consumption data is highlighted in a).

In addition to the individual demand data, the sum of the hourly electricity consumption of all households in each of the five Norwegian bidding areas, as shown in Fig. 1, are available from July 2019 to July 2022. Since the number of households, i.e., electricity meters, are available in the dataset, the hourly consumption of an average household can be calculated as illustrated in Fig. 5.

#### 3.1.4 Electricity spot prices

**Fig. 6:**
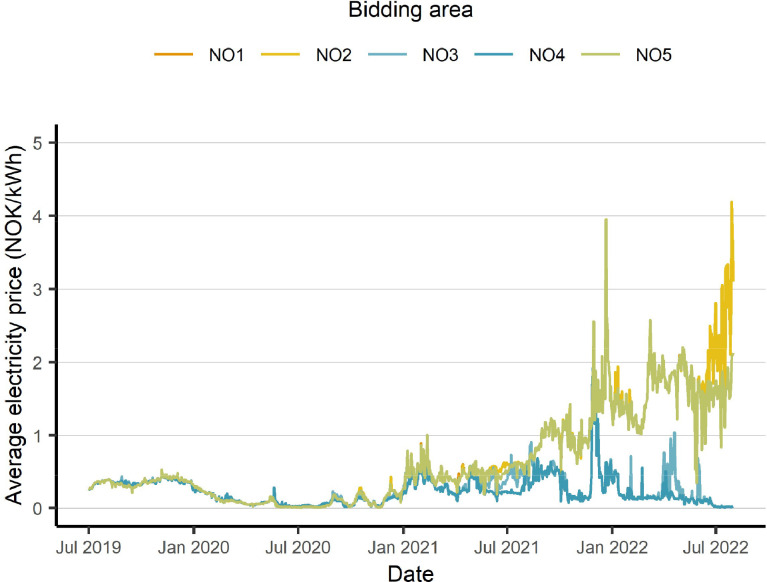
Electricity prices per bidding area for the observation period illustrated as daily average values based on the hourly values from the dataset.

The hourly electricity price for each of the bidding zones are added to the dataset since most of the Norwegian households have electricity tariffs tied to the spot price from the day-ahead market. The extraordinary increase in electricity prices in the bidding areas NO1, NO2 and NO5, which all are in southern Norway, can be seen in Fig. 6.

### 3.2 Structure of table files

The dataset contains the following comma-separated files, which are described in the following sections:

• households.csv
• survey.csv
• answers.csv
• demand.csv
• demand_areas.csv
• prices.csv

#### 3.2.1 Households.csv

This file contains an overview of all 4,446 households which answered the survey with information about their location of residence and whether electricity consumption data are available. Each participant has a unique identifier, also used in *answers.csv* and *demand.csv*. A detailed description of the columns in the file is provided in Table 2.

**Table 2:** Description of the table structure in households.csv.

Column name	Possible values	Short description
ID	18, …, 9085	Unique identifier for each household
City	Asker, Bergen, Bærum, Lillestrøm, Oslo, Tromsø, Trondheim	Name of the household's city
Region	Bergen, Oslo, Tromsø, Trondheim	Name of the household's region
Price_area	NO1, NO3, NO4, NO5	Name of the household's bidding area
Demand_data	Yes, No	Specifies if electricity demand data are available for this household

#### 3.2.2 Survey.csv

All households in the dataset answered a survey, and this file contains all questions and possible answer options, both in the original Norwegian version and translated into English. A detailed description of the columns in the file is provided in Table 3.

**Table 3:** Description of the table structure in survey.csv.

Column name	Possible values	Short description
Question_ID	Q_Age, Q_Gender, Q_City, Q1, …, Q32	Unique identifier for each question
Option_number	1, …, 10, None	Identifier for each answer option *None*: Numerical question with no options
Question	Text	Original Norwegian version of the question
Option	Text or NA	Original Norwegian version of the answer options
Question_translation	Text	English translation of the question
Option_translation	Text or NA	English translation of the answer options

#### 3.2.3 Answers.csv

This file contains the answers to the survey of each of the 4,446 households. It is organised in a matrix with households in rows and answers in columns. A detailed description of the columns in the file is provided in Table 4.

**Table 4:** Description of the table structure in answers.csv.

Column name	Possible values	Short description
ID	18, …, 9085	Unique identifier for each household
Q_Age, Q_Gender, Q_City, Q1, …, Q32	1, …, 10, NA	Answers to the question with the same *Question_ID* as the column name. The number represents either the *Option_number* or a numerical answer. The question and option text can be found in *survey.csv.* *NA*: Respondent did not answer this question or did not choose that option (if multiple choice answer)

#### 3.2.4 Demand.csv

This file contains the hourly electricity consumption data for households which consented to share their demand data. A detailed description of the columns in the file is provided in Table 5.

**Table 5:** Description of the table structure in demand.csv.

Column name	Possible values	Short description
ID	18, …, 9085	Unique identifier for each household
Date	yyyy-mm-dd	Date of observation
Hour	1, …, 24	Hour of the day
Demand_kWh	0 – 19.64	Electricity consumption in kWh/h

#### 3.2.5 Demand_areas.csv

This file contains the sum of the hourly electricity consumption of all households in each of the five Norwegian bidding areas. A detailed description of the columns in the file is provided in Table 6.

**Table 6:** Description of the table structure in demand_areas.csv.

Column name	Possible values	Short description
Price_area	NO1, NO2, NO3, NO4, NO5	Name of the bidding area that the aggregated consumption data are from
Date	yyyy-mm-dd	Date of observation
Hour	1, …, 24	Hour of the day
Demand_MWh	149 - 3935	Electricity consumption in MWh/h
Meter_count	238,279 – 1,072,105	The number of electricity consumption meters on which the aggregated data are based
Demand_kWh_avg	0.56 – 4.16	Mean electricity consumption per meter in kWh/h

#### 3.2.6 Prices.csv

This file contains the hourly day-ahead prices of electricity for each of the five Norwegian bidding areas. A detailed description of the columns in the file is provided in Table 7.

**Table 7:** Description of the table structure in prices.csv.

Column name	Possible values	Short description
Price_area	NO1, NO2, NO3, NO4, NO5	Name of the bidding area that the aggregated consumption data are from
Date	yyyy-mm-dd	Date of observation
Hour	1, …, 24	Hour of the day
Price_NOK_MWh	-19.72, – 6538.94	Electricity consumption in MWh/h
Price_NOK_kWh	-0.02 – 6.54	The number of electricity consumption meters on which the aggregated data are based

## 4 Experimental Design, Materials and Methods

The survey answers, and the hourly data on electricity consumption and prices, were collected with different methods. The collection methods are described briefly in the following sections.

### 4.1 Survey answers

The questions were derived from a similar survey conducted with Norwegian households one year prior, which was published and described in Ref. [1]. Our survey was conducted from 30 March to 3 May 2022, with households that were part of Ipsos' web panel. The household sample was limited to four Norwegian regions, thus missing one bidding area, as depicted in Fig. 1. It was assumed that the answers from the households in Bergen and Oslo were representative of the missing bidding area NO2, given the comparable electricity prices and climate conditions. A list of the survey questions is provided in Table 8 to demonstrate the available information per household. Nearly all questions were multiple-choice format, and the answer options can be found in survey.csv.

**Table 8:** List of the survey questions.

Age?
Gender?
City?
Did you monitor your power consumption this winter?
How did you acquire information about your power consumption?
Why did you not monitor your consumption?
Did you monitor the variation in electricity prices from day to day and hour to hour this winter?
How did you acquire information about the electricity prices?
Why did you not monitor the electricity prices?
Did you take any measures to decrease or move power consumption from hours with high prices this winter?
Which measure did you implement?
Do you know how much the household has saved on the power bill as a result of the measures?
About how much has the household saved per month this winter as a result of the measures?
Do you feel that the measures you implemented were worth the savings on the power bill?
Why did you not take any measures?
What motivates you to reduce your power consumption in high price hours?
How much do you agree or disagree with the following statement? People who adjust their power consumption based on price should be able to save on their power bill.
Would you or have you used a free information service that alerts you of high price hours the following day?
How many persons does your household consist of, including yourself?
What age are the inhabitants of you household, including yourself?
Imagine you can buy smart devices for 5,000 NOK that will reduce your power bill by automatically shifting parts of you consumption away from high price hours - without reducing comfort. How much would you have to save every year to do it?
How many weekdays (Mon-Fri) on average was there anyone home in the day (9 a.m. to 4 p.m.) this winter (Nov-Mar)?
What is the highest education in the household?
What is the combined gross income of the household?
What type of residence do you live in?
How big is the residence?
Do you own the residence?
Do you have a rental unit in the residence?
Does the rental unit have its own power meter?
How is the residence heated?
How is the tap water heated?
Do you own one or more electric car that is at least sometimes charged at home?
How is the car or cars normally charged?
Do you control the car charging to avoid hours with high prices?
What type of power contract do you have?

### 4.2 Hourly data

Hourly electricity consumption data and electricity spot prices were collected. To mitigate complications arising from daylight-savings time switches, each day in the dataset has 24 hours. The springtime missing hour was calculated as the average of values from the preceding and succeeding hours. The extra hour in autumn was dealt with by omitting the second hour of the doubled hours.

#### 4.2.1 Individual electricity consumption

Historical metered consumption data were collected from the households. It is important to note that these data were not influenced by the survey because, at the time of electricity consumption, the households were unaware that their data would be shared. As part of the survey, we included a question about whether the household consented to share their electricity consumption data, of which 67% or 3,011 households consented.

The electricity data were subsequently extracted by the customers' respective distribution grid companies covering October 2020 to March 2022 and anonymised by assigning each customer a randomised identifier. To ensure privacy, this identifier was connected to the electricity consumption data and survey responses. Consumption data were only received from the grid companies in the regions of Oslo and Bergen. The amount of data was further reduced due to grid companies' inability to match each household that consented to their customer database. This occurred, since the information given in the survey, i.e., name and address, was not always sufficient to identify a unique customer. For this reason, the number of households received was 1,609.

Many households had missing consumption values or several values for some hours. After the data quality was checked and all households with data issues had been removed, the number of the remaining households was 1,161.

**Fig. 7:**
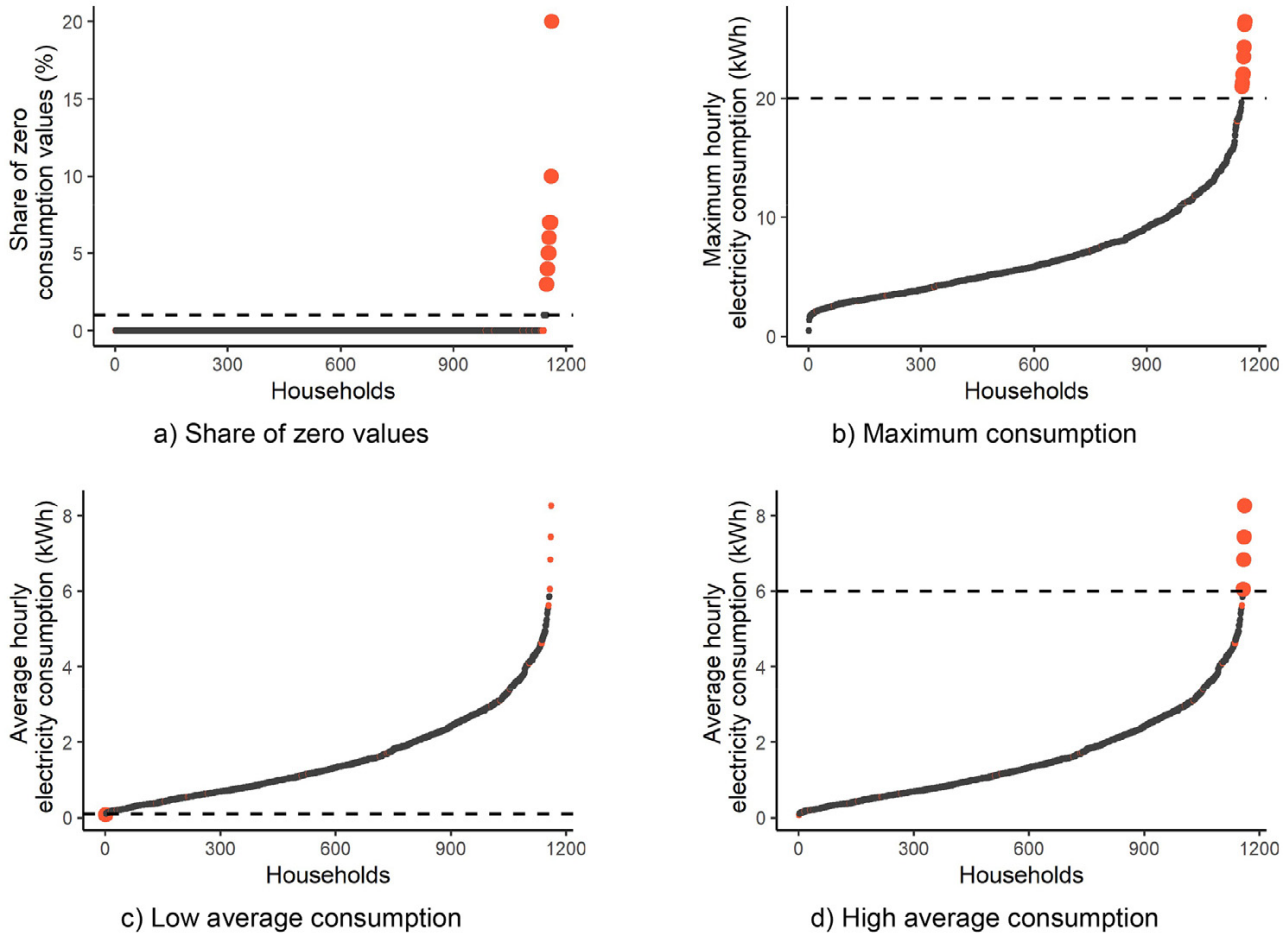
Threshold values (dashed line) and excluded households (red dots) for the four exclusion criteria.

In addition, households with unusual consumption values were excluded from the dataset as visualised in Fig. 7. Four criteria identified the outliers based on three consumption characteristics, i.e., the share of zero values, maximum consumption, and average consumption. The specification of the threshold values was performed graphically, and households with a share of zero values higher than 1%, maximum consumption above 20 kWh/h, or average consumption above 6 kWh/h or below 0.1 kWh/h were excluded.

In total, 25 households were identified as outliers and excluded. Table 9 summarises the exclusion reasons for the two regions for which the data were collected.

**Table 9:** Excluded households per region and exclusion criteria which led to exclusion.

	Oslo	Bergen	Sum
In total	18	7	25
Zero > 1%	10	4	14
Average > 6 kWh/h	3	1	4
Average < 0.1 kWh	0	1	1
Max > 20 kWh/h	7	2	9

After excluding outliers, the electricity consumption data of 1,136 households were included in the final dataset, as summarised in Table 10.

**Table 10:** Availability of individual electricity consumption data per region.

Region	Consent	Data received	Correct data	Final dataset
Oslo	1,556	1,025	806	788
Bergen	678	584	355	348
Trondheim	528	0	0	0
Tromsø	249	0	0	0
Sum	3,011	1,609	1,161	1,136

#### 4.2.2 Aggregated electricity consumption

Hourly aggregated electricity consumption data of households in each of the five bidding areas were collected for the period July 2019 to July 2022. All data were acquired from the national electricity consumption database Elhub^2^ which collects, stores, and aggregates hourly consumption data for all electricity consumer groups in Norway.

#### 4.2.3 Electricity spot prices

Hourly electricity spot prices for each of the five bidding areas were included in the dataset in the Norwegian currency NOK for the period July 2019 to July 2022. The prices were retrieved from Nordpool and are openly available in EUR from the ENTSOe Transparency platform^3^. Daily currency exchange rates between EUR and NOK can be downloaded from the website of Norges Bank, the Norwegian central bank^4^.

## Ethics Statement

All households gave informed consent to share their electricity consumption data and to allow for a combination with the survey answers to publish anonymised data.

## Declaration of generative AI and AI-assisted technologies in the writing process

During the preparation of this work the authors used ChatGPT and Grammarly to improve readability and language in general. After using these tools, the authors reviewed and edited the content as needed and take full responsibility for the content of the publication.

## CRediT authorship contribution statement

**Matthias Hofmann:** Conceptualization, Methodology, Investigation, Data curation, Writing – original draft, Writing – review & editing, Visualization, Supervision, Project administration, Funding acquisition. **Sigurd Bjarghov:** Data curation, Software, Writing – original draft, Writing – review & editing. **Stian Nessa:** Data curation, Software, Writing – original draft.

## Data Availability

Norwegian hourly residential electricity demand data with consumer characteristics during the European energy crisis (Original data) (Zotero)

## Footnotes

1 https://www.nordpoolgroup.com/en/maps/#/nordic.

2 https://elhub.no/. Distribution grid companies, retailers, and customers can access their customers and their own electricity consumption data, respectively.

3 https://transparency.entsoe.eu/transmission-domain/r2/dayAheadPrices/show.

4 https://www.norges-bank.no/en/topics/Statistics/exchange_rates/?tab=currency&id=EUR.
